# Near-Infrared Spectral Characteristic Extraction and Qualitative Analysis Method for Complex Multi-Component Mixtures Based on TRPCA-SVM

**DOI:** 10.3390/s22041654

**Published:** 2022-02-20

**Authors:** Guiyu Zhang, Xianguo Tuo, Shuang Zhai, Xuemei Zhu, Lin Luo, Xianglin Zeng

**Affiliations:** 1 School of Information Engineering, Southwest University of Science and Technology, No. 59 Qinglong Road, Mianyang 621010, China; zhangguiyu@suse.edu.cn; 2 School of Automation & Information Engineering, Sichuan University of Science & Engineering, No. 1 Baita Road, Yibin 644000, China; 31908110401@stu.suse.edu.cn (S.Z.); 320085404237@stu.suse.edu.cn (X.Z.); 31908521041@stu.suse.edu.cn (L.L.); 31808521022@stu.suse.edu.cn (X.Z.); 3 Artificial Intelligence Key Laboratory of Sichuan Province, No. 1 Baita Road, Yibin 644000, China

**Keywords:** multi-component, near-infrared spectroscopy, characteristic extraction, difference spectrum, qualitative analysis

## Abstract

Quality identification of multi-component mixtures is essential for production process control. Artificial sensory evaluation is a conventional quality evaluation method of multi-component mixture, which is easily affected by human subjective factors, and its results are inaccurate and unstable. This study developed a near-infrared (NIR) spectral characteristic extraction method based on a three-dimensional analysis space and establishes a high-accuracy qualitative identification model. First, the Norris derivative filtering algorithm was used in the pre-processing of the NIR spectrum to obtain a smooth main absorption peak. Then, the third-order tensor robust principal component analysis (TRPCA) algorithm was used for characteristic extraction, which effectively reduced the dimensionality of the raw NIR spectral data. Finally, on this basis, a qualitative identification model based on support vector machines (SVM) was constructed, and the classification accuracy reached 98.94%. Therefore, it is possible to develop a non-destructive, rapid qualitative detection system based on NIR spectroscopy to mine the subtle differences between classes and to use low-dimensional characteristic wavebands to detect the quality of complex multi-component mixtures. This method can be a key component of automatic quality control in the production of multi-component products.

## 1. Introduction

The single-component quantitative detection technology of substances is mature, such as in the direct measurement of electrochemical sensors and the use of spectroscopy combined with chemometrics to establish quantitative models [1,2,3,4]. Currently, multi-component quantitative and qualitative analysis of complex systems has become the focus of research. The application scope of multi-component analysis includes the food, pharmaceutical, medical, petrochemical, and environmental monitoring fields. For simultaneous detection of coexisting components, traditional chemical analysis methods require pretreatment, such as component separation, which is time-consuming and labor-intensive, and which has low detection accuracy. With the development of computer science and technology, spectroscopy technology has been widely used in multi-component analysis. As early as the 1980s, some scholars used visible spectroscopy, ultraviolet spectroscopy, and infrared spectroscopy to detect metal ions and RNA mixtures, and they have carried out corrections of background noise in the spectrum and quantitative analyses of multi-components [5,6]. With the continuous deepening of research, multi-component quantitative analysis of complex bodies based on spectral detection technology has been rapidly developed [7,8,9]. The quantitative analysis method takes the component content and spectral data as the training set and uses chemometric algorithms to establish the predictive model to realize rapid and non-destructive quantitative detection [10,11,12]. The content of the detected component must be greater than the minimum detection limit of the quantitative analysis instrument, and trace components cannot be quantitatively detected. However, the class of the complex mixture is affected by the interaction of all its components. Therefore, the quantitative detection of only some of the components cannot accurately facilitate qualitative analysis. In view of this, this article selected multi-component distilled liquor as an example and carried out rapid and non-destructive qualitative analyses of it. Liquor is extracted by distilling fermented brewing materials to extract alcohol and trace substances. Flavor substances include alcohol, acids, esters, aldehydes, ketones, phenols, higher alcohols, and polyols, and more than one thousand different compounds [13,14,15].

In addition to water and alcohol, other compounds in the liquor have many categories and low contents. The contents of some compounds are lower than the detection limit of current detection equipment, such as mass spectrometry, spectrophotometry, high-performance liquid chromatography and gas chromatography. The application of these traditional detection methods in rapid detection and process control is limited due to their complicated preprocessing procedures. At present, it is necessary to rely on artificial sensory evaluation, but the unavoidable defects, such as strong subjectivity and poor reproducibility, still make it impossible to obtain an accurate quantitative evaluation result. Therefore, we should apply multi-disciplinary knowledge to make the production technology of liquor and other multi-component products standardized, scientific, and systematized [16].

Near-infrared (NIR) spectroscopy analysis is a nondestructive detection and rapid analysis method and has been widely applied to quantitative detection and qualitative identification. In this paper, NIR spectroscopy was used for the qualitative identification of liquor. The most prominent absorption bands occurring in the NIR region are related to overtones and combinations of fundamental vibrations of the –CH, –NH, –OH, and –SH functional groups. By scanning liquor samples using NIR spectroscopy, the characteristic information of the hydrogen-containing groups of organic molecules in the sample can be obtained. The absorption of molecular frequency doubles, and the combined frequency is weak, and NIR light can penetrate deep into the sample [17]. Therefore, NIR is suitable for cases where the compound content is below the detection limit and can better reflect the coordination effect of multiple components of liquor quality.

In NIR analysis and detection, principal component analysis (PCA), multivariable linear regression (MLR), partial least-squares (PLS) and partial least squares discriminant analysis (PLS-DA) are commonly used to establish quantitative and qualitative models [18]. PLS is mainly used for quantitative analysis [11,19,20]. NIR spectral data have high-dimensional characteristics, and training a prediction model based on the full spectrum has a high computational cost and low prediction accuracy. Therefore, the PCA algorithm is often used for characteristic extraction to reduce the NIR spectral data’s dimensionality [21,22,23]. Furthermore, PLS-DA, support vector machine (SVM), neural network (NN), Soft Independent Modeling of Class Analogy (SIMCA) and other pattern recognition algorithms are used to construct classification models to ensure quality and authenticity [23,24,25,26,27]. 

To date, although these technologies have the potential for quantitative and qualitative classification, they still cannot replace sensory evaluation for complex multi-component mixtures, such as liquor. The NIR spectral data of a sample generally contain three-dimensional attributes of the sample, spectrum, and class. However, PCA-based characteristic extraction can only process data in two-dimensional space, ignoring the implicit relationships in three-dimensional space. As a result, subtle differences between different classes are overwhelmed [28], which affects the accuracy of prediction. Conventional dimensionality reduction methods transform the data into a new coordinate system, where each new variable is a linear combination of the original variables. It can be seen that the new variable reflects the comprehensive effect of the dimensionality of the original data [29]. In this study, taking liquor as an example, the tensor robust principal component analysis (TRPCA) algorithm was used to mine the characteristic information of the NIR spectra among different classes in the three-dimensional space, and the spectral data composed of a few characteristic wavenumbers were retained. Therefore, the main characteristics of the sample can be obtained only by detecting the spectrum of the characteristic wavenumber band. The TRPCA algorithm is a key technique for handling high-dimensional datasets and aims to recover low-rank and sparse components accurately. Using the kernel norm, the TRPCA problem is solved with convex programming. It is often used in image noise reduction and image restoration research [30,31,32,33]. However, no study has reported the use of the TRPCA algorithm for detecting multi-component mixtures. 

On the basis of characteristic extraction, the pattern recognition algorithm was used to establish a classification model. We hope that this method can promote the rapid, non-destructive, and accurate quality classification of the liquor industry. It can also be applied to the qualitative identification of multi-component products such as food, traditional Chinese medicine, and chemicals.

## 2. Materials and Methods

### 2.1. Liquor Samples

The liquor flavor system is complex and huge, and the flavor contribution and influence mechanisms of most components have not been studied clearly [14,34]. The Luzhou-flavored liquor was selected, and the samples were collected from a well-known winery in Sichuan Province. The samples were identified by professional tasters, and samples of five quality levels were selected for the experimental study. The sample sets were marked as five grades, I, II, III, IV, and V, each containing 150 samples (Table 1).

### 2.2. NIR Spectral Data Acquisition

The NIR spectral data of liquor samples were measured by a MATRIX-F Fourier transform near-infrared (FT-NIR) spectrometer, operating in transmittance mode. Before measurement, the spectrometer was preheated for 1 h, the test environment temperature was 20 °C, and the relative air humidity was <80%RH. The sample was scanned in the wavenumber range of 12,500~4000 cm^−1^, with a resolution of 4 cm^−1^. The dimensionality of each grade sample and sample set is shown in Table 1. Each sample was scanned 32 times, and the average spectrum was automatically taken. The time taken for measuring a sample was about 18 s.

### 2.3. Preprocessing of Spectral Data

The preprocessing of the NIR spectrum is an important step before sample analysis. It is used to remove noise such as random noise and baseline drift, while retaining as much useful information as possible. The derivative preprocessing algorithm can eliminate the influence of baseline drift and smooth background interference and distinguish overlapping spectral peaks. However, the derivative method will increase the noise in the spectrum. Therefore, the Norris derivative filtering (NDF) method was used to preprocess the spectral data. Before derivative preprocessing, smoothing preprocessing was performed to eliminate noise interference. The smoothing preprocessing adopted the van average method. First, the spectrum was divided into several equal fragments, and then each equal fragment was replaced by the average absorbance. The smoothed spectrum was calculated by applying the following equation:

(1) $${\bar{x}}_{i}=\frac{\sum_{j=1}^{N}x_{(i-1)N+j}}{N}$$

where ${\bar{x}}_{i}$ is the average absorbance of corresponding equal fragment, *x*_(*i*−1)*N+j*_ is the *j*-th absorbance value in the *i*-th fragment, and *N* is the smooth window size.

The direct difference method was used to calculate the first derivative of the discrete spectrum. The calculation equation for the first derivative was as follows:

(2) $$x_{k}^{\prime }=\frac{x_{k+g}-x_{k-g}}{2g}$$

where *k* is the wavenumber of discrete spectrum, and *g* is the width of first derivative.

Through the Norris derivative filtering method, smooth and high-signal-to-noise ratio NIR spectra were obtained, which were used to extract the characteristic wavenumbers.

### 2.4. NIR Differential Characteristic Expression Spectrum Extraction

**Figure 1:**
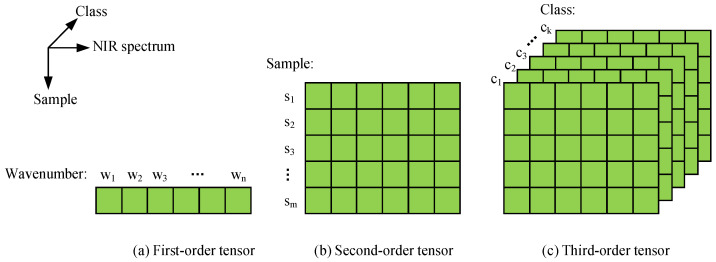
Third-order tensor matrix schematic diagram of NIR spectra.

Characteristic extraction was performed after the preprocessing of the NIR spectrum to achieve the purpose of reducing the dimensionality of the spectral data. Commonly used dimensionality reduction algorithms usually map high-dimensional data onto a two-dimensional space for analysis, such as PCA, linear discriminant analysis (LDA) and other algorithms. The above algorithms ignore the structural characteristics of the raw data. The NIR spectrum of a liquor sample included three dimensional attributes—the first dimension represented the sample, the second dimension represented the NIR spectral data, and the third dimension represented the sample class. Among them, the sample class was regarded as the structural characteristic. For the three-dimensional attributes, this paper adopted the third-order TRPCA algorithm. The algorithm was built in three-dimensional space (Figure 1).

The NIR spectra of liquor have strong similarity; thus, the spectral matrixes are low-rank matrixes. We defined the third-order tensor of the NIR spectral data as *D* and decomposed it into a low-rank tensor *A* and a sparse tensor *E*. The objective function was as follows:

(3) $$\underset{M,P}{\min}{\left\|A\right\|}_{*}+\eta {\left\|E\right\|}_{1},\mathrm{subject}\;\mathrm{to}\;D=A+E$$

where ${\left\|A\right\|}_{*}$ is the kernel norm of matrix *A*, which is equal to the sum of singular values of A, ${\left\|E\right\|}_{1}$ is the norm of matrix *E*, which is equal to the sum of absolute values of all items in E, and η is the weight parameter. η was calculated by applying the following equation:

(4) $$\eta =1/\sqrt{\max(w,s)c}$$

where *w*, *s* and *c* represent the size of the third-order tensor *A*: *w* × *s* × *c*.

In this paper, the alternating direction multiplier method was used to solve the convex optimization problem of the objective function, and the Lagrange multiplier was substituted into Formula (3). The Lagrange alternating direction multiplier method is shown in the following equation:

(5) $$L(A,E,y,\mu )={\left\|A\right\|}_{*}+\lambda {\left\|E\right\|}_{1}+y^{T}(A+E-D)+\mu /2{\left\|A+E-D\right\|}_{F}^{2}$$

where *μ* is a scalar parameter, *y* is a Lagrangian multiplier, and ${\left\|•\right\|}_{F}$ is the Frobenius norm.

**Figure 2:**
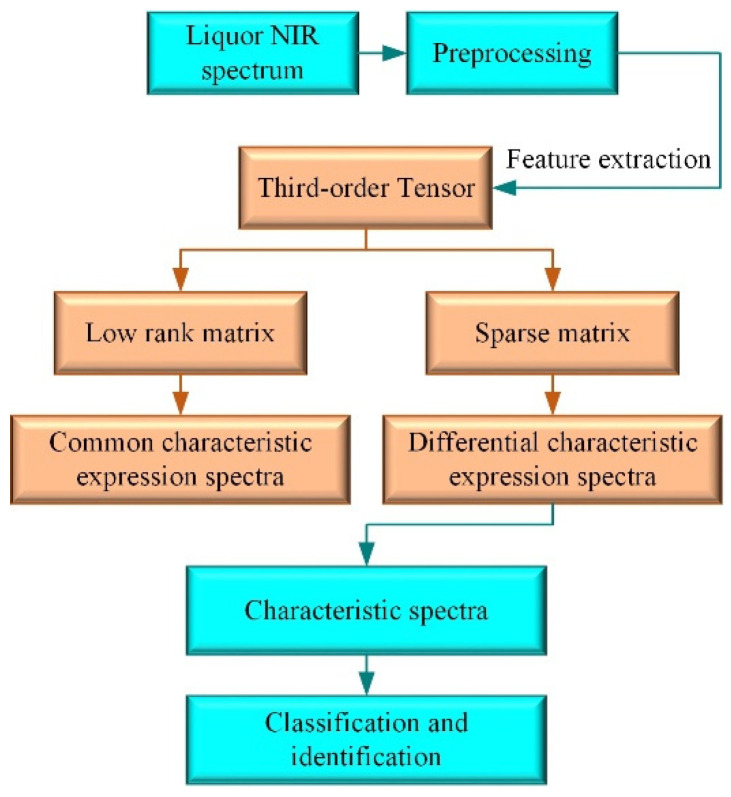
An illustration of NIR spectrum processing sequences used for liquor identification.

The low-rank tensor and sparse tensor were obtained through the above third-order TRPCA. The low-rank tensor and sparse tensor were used to extract the spectra containing common and differential characteristics, respectively. The differential characteristic-expressing spectra were used for the qualitative identification of liquor (Figure 2). The purpose of characteristic extraction and dimensionality reduction was achieved.

## 3. Results and Discussion

### 3.1. Preprocessing of NIR Spectrum Based on Norris Derivative Method

**Figure 3:**
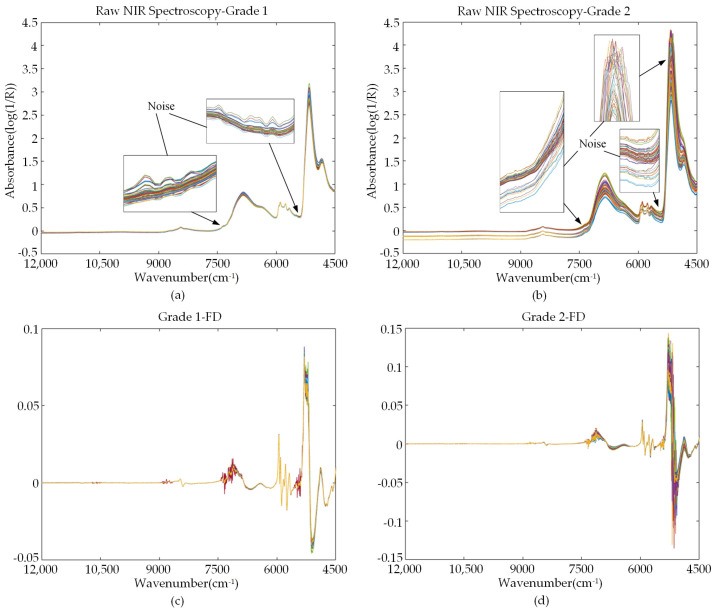
(**a**,**b**) Raw NIR spectra of sample sets I and II; (**c**,**d**) first derivative preprocessed NIR spectra of sample sets I and II.

Figure 3 shows the raw NIR spectra of five grades of liquor samples and the NIR spectra preprocessed by the first derivative. The raw NIR spectrogram shows that there were noise signals in the spectra (Figure 3a,b, taking sample sets I and II as examples). The noise signals are more obvious in the bands of 7500–6900, 5600–5400, and 5400–5000 cm^−1^. Figure 3c,d shows that after the derivative preprocessing of the raw spectra, the amplitudes of the spectral curves were significantly reduced, and the amplitudes of these curves were only between approximately −0.15 and 0.15. Obviously, the influence of noises was amplified, which affected the difference analysis and characteristic extraction of samples.

Therefore, NDF was adopted to solve the problem of the noise caused by the derivative being amplified. Before derivative pretreatment of the raw NIR spectrum, smoothing pretreatment was carried out. NDF is a group of multi-mode spectral preprocessing algorithms based on multiple variable parameters. NDF contains three variable parameters: smoothing points, derivative order, and difference interval. The fusion of parameters with different functions can enhance the flexible vitality of the NIR spectrum and meet the individual needs of preprocessing of the diverse spectrum.

**Figure 4:**
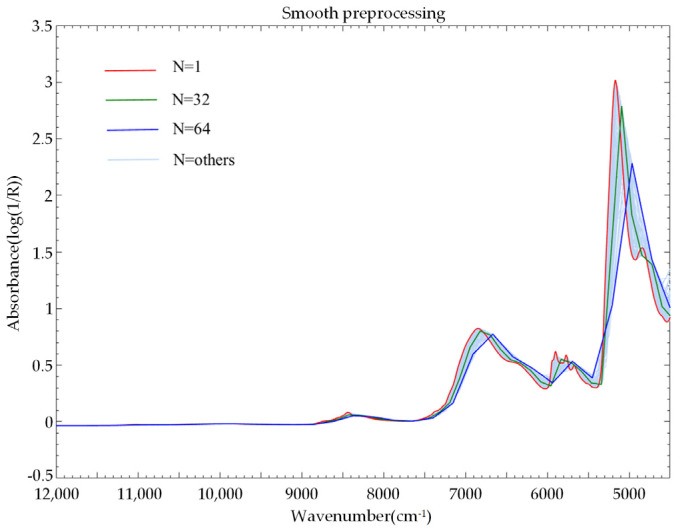
Evolution of the smoothing preprocessing spectrum with the smoothing window.

The smoothing pretreatment adopted the van smoothing method. Taking sample set II as an example, we selected the number of smoothing points N from 1 to 64, respectively, and obtained smoothed spectra (Figure 4). Among them, when the number of smoothing points was 1, it was the raw spectrum. As shown in the figure, as the number of smoothing points increased, the filtering effect also increased. The absorption peak shifted to the right, and the amplitude gradually decreased and became smoother. 

**Figure 5:**
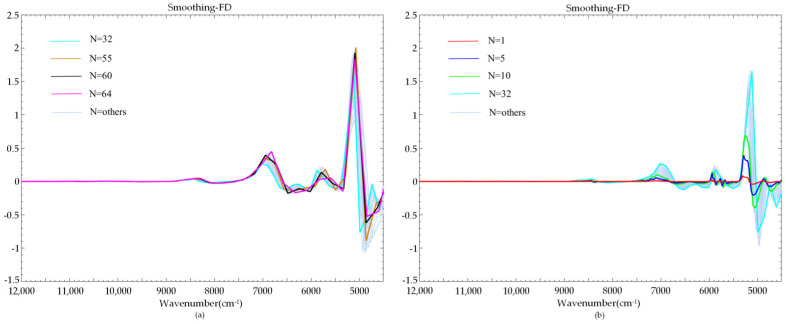
First derivative preprocessed spectra with different smoothing windows. (**a**) The smoothing window was between 1 and 32; (**b**) the smoothing window was between 32 and 64.

On the basis of smoothing preprocessing, first derivative preprocessing was carried out (Figure 5). As shown in Figure 5a, the smaller the smoothing window, the smaller and flatter the spectral amplitude. As the smoothing window increased, the characteristic peaks gradually became more prominent. As shown in Figure 5b, as the smoothing window continued to increase, the amplitude of the main absorption peak of the spectra preprocessed by the first derivative gradually reached the maximum. The absorption peaks with smaller amplitudes were gradually flattened, such as the 6000–5500 and 5300–4500 cm^−1^ wavenumber bands.

It can be seen that if the smoothing window of the NDF algorithm was too small, the characteristic peaks were not obvious, and they were easily affected by noise. If the smoothing window was too large, some of the absorption peaks disappeared, resulting in the loss of the characteristic information of the NIR spectrum. Therefore, further characteristic extraction was performed on the spectra after NDF preprocessing, and the best smooth preprocessing window size was determined through the prediction accuracy of the qualitative identification model.

### 3.2. Characteristic Spectrum Extraction Based on the Third-Order TRPCA

**Figure 6:**
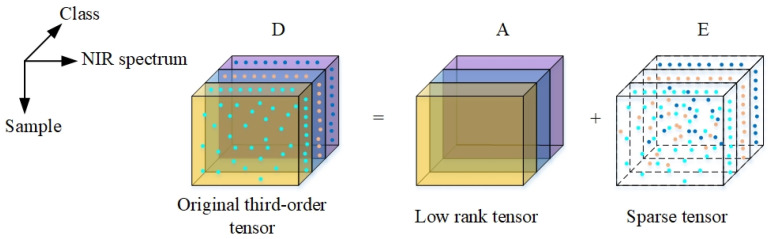
Third-order tensor robust principal component analysis model.

The characteristic peaks of the NIR spectra preprocessed by NDF were mainly concentrated in the wavenumber range of 8000–4500 cm^−1^ (Figure 5), and this wavenumber range was used as the target band of the NIR spectrum characteristic extraction research. Characteristic extraction used the third-order TRPCA, and the multi-view model is shown in Figure 6. In the three-dimensional analysis space, the NIR spectra were represented by a third-order tensor *D* and decomposed into a low-rank tensor *A* and a sparse tensor *E*.

**Figure 7:**
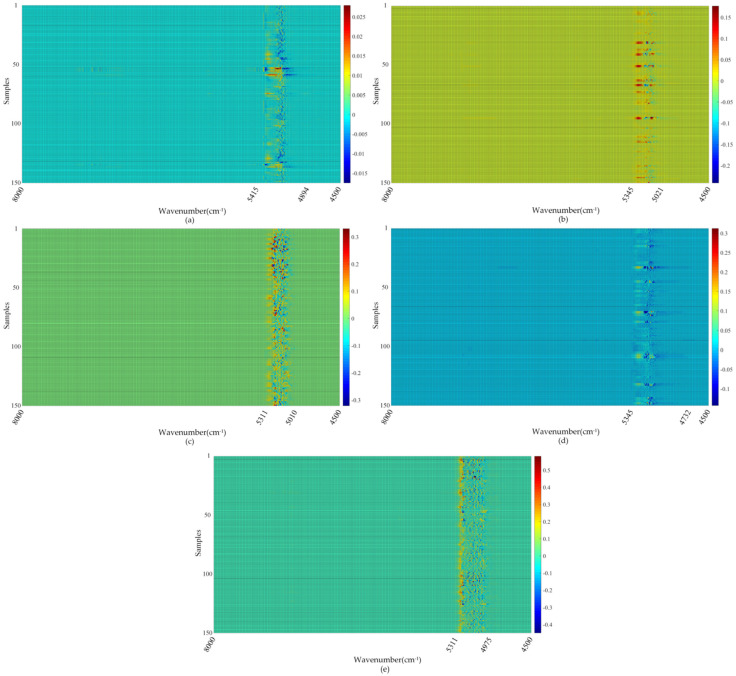
(**a**–**e**) The heat maps of the sparse matrices of five classes of liquor samples, respectively.

**Figure 8:**
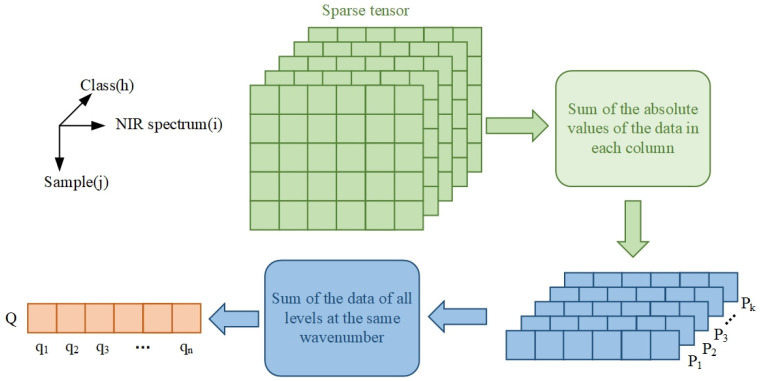
Characteristic spectrum extraction method based on sparse tensor.

The low-rank tensor mainly contained common expression characteristics, and the differential expression characteristics (scattered points in Figure 6) were mainly concentrated in the sparse tensor. The sparse tensor was used to extract the difference in characteristic information in the NIR spectra of different classes of liquor. The smoothing window was set to 23 as an example. Figure 7 shows the heat maps of the sparse matrices of five classes of liquor. The differential expression wavenumbers were concentrated in the bands of 5415–4894, 5345–5201, 5311–5010, 5345–4732, and 5311–4975 cm^−1^. On this basis, the sparse matrices were further analyzed (Figure 8).

**Figure 9:**
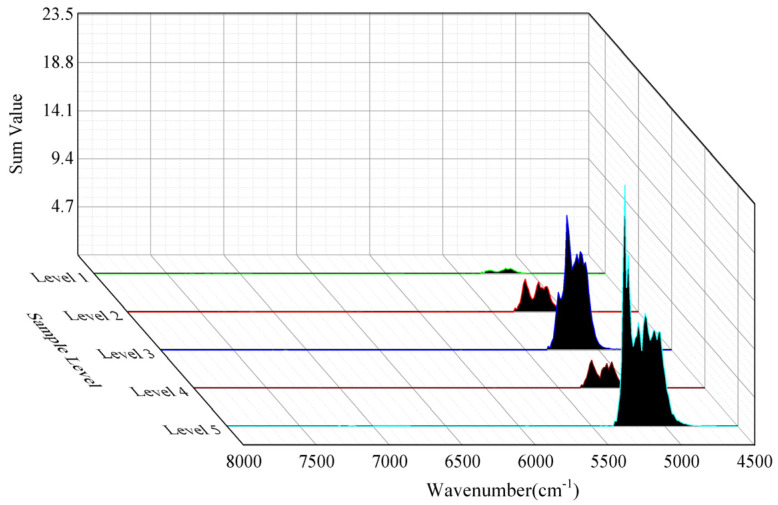
Difference spectrum distribution diagram of each level sample.

First, we calculated the absolute value of the data of each sparse matrix and summed each column:

(6) $$P_{h}=(p_{h1},p_{h2},\ldots ,p_{hn})$$

(7) $$p_{hi}=\sum_{j=1}^{m}\left|w_{ij}\right|$$

where *i* is the wavenumber of the NIR spectra, *j* is the number of samples in each class, *h* is the number of the class, *w_ij_* is the value of the sparse matrix element, *p_hi_* is the sum of the absolute values of the columns, and *P_h_* is the vector calculated by each sparse matrix. Figure 9 shows the distribution of vectors of each class.

**Figure 10:**
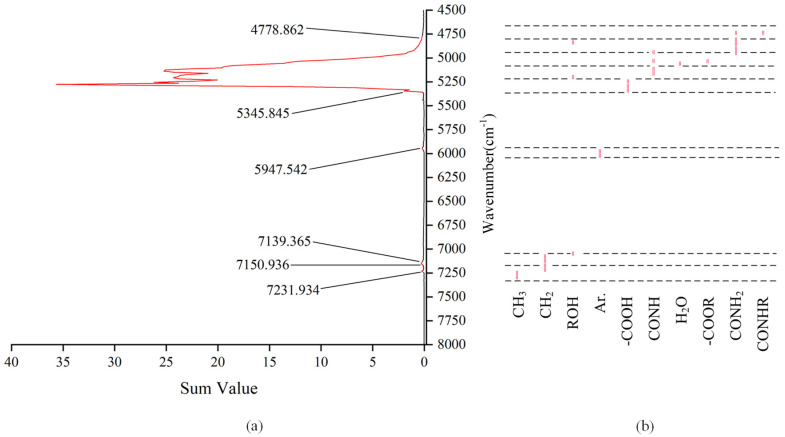
(**a**) Processing result of the sparse tensor of five classes of liquor; (**b**) main groups of the absorption band at the characteristic wavenumbers (“=”: the absorption position of the group).

Then, the element values of the same wavenumber in the five *P_h_* vectors were summed—the equation is as follows:

(8) $$q_{i}=\sum_{h=1}^{k}\left|{\bar{w}}_{ih}\right|$$

(9) $$Q=(q_{1},q_{2},\ldots ,q_{n})$$

where *q_i_* is the sum of all elements of the same wavenumber in *P_h_*, and *Q* is the new vector finally obtained (Figure 10). We arranged *q_i_* in descending order—the higher the ranking, the more significant the difference expression of the wavenumber, and the more likely they were to be the characteristic wavenumbers.

For each element in the vector *Q*, the filtering threshold was set to 0.2. The wavenumbers corresponding to the red curve in Figure 10a are the obtained characteristic wavenumbers. The absorption peaks at the characteristic wavenumbers constituted the characteristic spectrum. Table 2 shows the source of the main absorption band. According to Table 2, Figure 10b shows the main groups of the characteristic spectrum, including methyl (CH3), methylene (CH2), alcohol group (ROH), benzene ring (Ar.), carboxyl group (-COOH), ester group (-COOR), imide group (CONH), primary amide group (CONH_2_), secondary amide group (CONHR), and inorganic water (H_2_O). The analysis results show that the characteristic information of the NIR spectrum of liquor could interpret the coordination effect of the above groups.

### 3.3. Qualitative Identification Based on Characteristic Spectrum 

This study used a multi-class support vector machine (SVM) algorithm for qualitative analysis. SVM has strong generalization and global optimization abilities in solving the pattern recognition of nonlinear and high-dimensional datasets. NIR spectral data are not linearly separable in the original characteristic space. SVM mapped the original characteristic space to a high-dimensional space through a mapping function, and samples were classified in the new space. The objective function and constraint condition of SVM optimization are as follows:

(10) $$\begin{array}{l} \min\frac{1}{2}{\left\|\omega \right\|}^{2}+C\sum_{i=1}^{n}\xi (i)\\ s.t.y_{i}(\omega^{T}\Phi (x_{i})+b)\geq 1-\xi (i),i=1,2,\ldots ,n \end{array}$$

where *x_i_* is the observed variable of the sample, *y_i_* is the class label, *w* and *b* are the parameters of the classification hyperplane, Φ(*·*) is the mapping function, *ξ*(*i*) is the relaxation factor, and *C* is the penalty factor. The relaxation factor allows for classification errors in the hyperplane, and the penalty factor prevents the relaxation factor from being too large. When *C* is large, *ξ*(*i*) reduces to zero, which means that the tolerance for misclassification is low. On the contrary, the possibility of misclassification is greater. We used the kernel function to calculate the sample distance in the high-dimensional space and selected the following polynomial kernel function in Matlab R2018a:

(11) $$G(x_{i},x_{j})={\left(\gamma x_{i}^{T}x_{j}+r\right)}^{q}$$

where *x_i_* and *x_j_* are the observed variables of the sample, *q* is the highest degree of the polynomial kernel function, and *γ* and *r* are the kernel function parameters.

**Figure 11:**
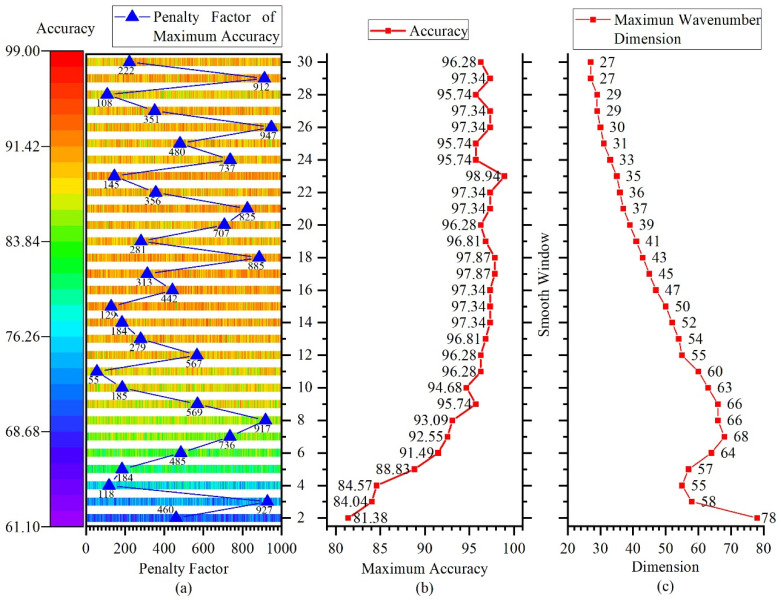
(**a**) Distribution of classification accuracy under different configurations of the smoothing window and penalty factor; (**b**) maximum classification accuracy of different smoothing windows; (**c**) characteristic wavenumber dimension of different smoothing windows.

This study tested different values for the smoothing window and penalty factor parameters of the Norris derivation method. The smoothing window testing range was from 2 to 20, and the penalty factor testing range was from 1 to 1000. The NIR spectral characteristic extraction and classification prediction results of liquor samples are shown in Figure 11.

From Figure 11a, it can be found that when the smoothing preprocessing window was small, the generalization ability of the prediction model was low. Combined with the aforementioned preprocessing analysis, it can be seen that the noise in the smoothing window had a significant impact on characteristic extraction. The larger the smoothing window, the smaller the influence of noise. As the size of the smoothing window increased, the generalization ability of the prediction model gradually increased. By adjusting the penalty factor, the optimal prediction model under each smoothing preprocessing window can be obtained. Figure 11b shows the highest prediction accuracy of the model with different smoothing preprocessing windows. When the smoothing preprocessing window was between 13 and 23, a higher and stable prediction accuracy could be obtained. Figure 11c shows the dimensionality of the characteristic wavenumber obtained through characteristic extraction under different smoothing preprocessing windows. The size of the smoothing window started at seven, and as the window increased, the dimensionality of the characteristic wavenumber gradually decreased.

The penalty factor reflected the tolerance to misclassification during the training process of the prediction model, but too large a penalty factor could easily lead to overfitting of the prediction model. Figure 11a marks the penalty factor of the optimal prediction model under each smoothing preprocessing window. When the smoothing window was 23, a relatively small penalty factor was selected at 145, and the dimensionality of the NIR spectrum after characteristic extraction was 35, which can achieve the highest prediction accuracy of 98.94%. This shows that the characteristic extraction algorithm and prediction model studied in this paper could effectively extract the characteristic information of high-dimensional NIR spectrum and achieve high-accuracy qualitative analysis.

## 4. Conclusions

In this paper, NIR transmittance spectroscopy was used as the detection method of a multi-component mixture, and an effective spectral characteristic extraction and qualitative analysis method was obtained. This article focused on liquor with complex multi-component characteristics as the research object. First, the Norris derivation method was used to remove interference signals and obtained the smooth main absorption peaks. Characteristic extraction algorithm was based on a three-dimensional analysis space and was able to mine subtle differences in the spectrum. Through third-order TRPCA, the characteristic spectrum was obtained and effectively reduced the dimensionality of the original NIR spectrum. Finally, the characteristic spectra were used as the training sets to build the SVM qualitative classification model. The research results show that a prediction accuracy of 98.94% could be obtained using spectral data in characteristic wavenumber bands. Our results suggest that TRPCA can accurately extract the characteristic information of the NIR spectrum, and the optimal prediction model can realize the accurate identification of liquor. In the future, TPRCA-SVM qualitative identification technology based on NIR spectroscopy can be used for the rapid qualitative detection of multi-component mixtures, such as food, traditional Chinese medicine, and chemicals. This method can also be developed into a key automated method for quality control.

## Figures and Tables

**Table 1:** Sample set information identified by professional tasters.

Sample Grade	Number of Samples	NIR Spectral Data Sampling Points	Dimensionality	Dimensionality of Sample Sets
I	150	2125	2125 × 150	2125 × 150 × 5
II	150	2125	2125 × 150	
III	150	2125	2125 × 150	
IV	150	2125	2125 × 150	
V	150	2125	2125 × 150	

**Table 2:** Source of the main absorption band at the characteristic wavenumbers of the NIR spectrum [35].

Wavenumber/cm^−1^	Vibration Mode	Structure	Wavenumber/cm^−1^	Vibration Mode	Structure
7353	2× C-H str. + C-H def.	CH_3_	5128	3× C=O str.	-COOR
7168	2× C-H str. + C-H def.	CH_2_	5102	N-H asym.str. + amide II	CONH
7092	2× O-H str.	ROH	5000	N-H sym.str. + amide II	CONH_2_, CONHR
5935	2× C-H str.	Ar.	4926	3× C=O str.	CONH_2_
5263	3× C=O str.	-COOH	4878	N-H asym.str. + amide II	CONH_2_
5241	2× O-H str.	ROH	4808	O-H str. + O-H def.	ROH
5208	2× C=O str.	CONH	4739	N-H sym.str. + amide III	CONH_2_, CONHR
5155	O-H str. + O-H def.	H_2_O			

Symbol description in the table: “2×”, “3×”: the double and triple frequency of the fundamental frequency. “str.”, “def.”: stretching vibration and deformation vibration. “sym”, “asym.”: symmetrical vibration and asymmetrical vibration. “amide I (II, III)”: the different coupling modes of the carbonyl group and the amine group in the amide molecule.

## Data Availability

The data presented in this study are available on request from the corresponding author. The data are not publicly available due to the components involved in the liquor.

## References

[1] Lyu X., Hamedpour V., Sasaki Y., Zhang Z., Minami T. (2021). 96-Well Microtiter Plate Made of Paper: A Printed Chemosensor Array for Quantitative Detection of Saccharides. Anal. Chem..

[2] Shiraishi Y., Ichimura C., Hirai T. (2007). A quinoline–polyamine conjugate as a fluorescent chemosensor for quantitative detection of Zn(II) in water. Tetrahedron Lett..

[3] Dallaire F., Picot F., Tremblay J.P., Sheehy G., Lemoine É., Agarwal R., Kadoury S., Trudel D., Lesage F., Petrecca K. (2020). Quantitative spectral quality assessment technique validated using intraoperative in vivo Raman spectroscopy measurements. J. Biomed. Opt..

[4] Zhao M.T., Zhang D.W., Zheng L.L., Condliffe O., Kang Y. (2020). Rapid quantitative detection of mineral oil contamination in vegetable oil by near-infrared spectroscopy. Chin. Opt. Lett..

[5] Liang Y.Z., Kvalheim O.M., Keller H.R., Massart D.L., Kiechle P., Erni F. (1992). Heuristic evolving latent projections resolving two-way multicomponent data. 2. Detection and resolution of minor constituents. Anal. Chem..

[6] Otto M., Wegscheider W. (1985). Spectrophotometric multicomponent analysis applied to trace metal determinations. Anal. Chem..

[7] Saito Y., Kakuda K., Yokoyama M., Kubota T., Tomida T., Park H.-D. (2016). Design and daytime performance of laser-induced fluorescence spectrum lidar for simultaneous detection of multiple components, dissolved organic matter, phycocyanin, and chlorophyll in river water. Appl. Opt..

[8] Rodriguez-Saona L.E., Khambaty F.M., Fry F.S., Dubois J., Calvey E.M. (2004). Detection and Identification of Bacteria in a Juice Matrix with Fourier Transform–Near Infrared Spectroscopy and Multivariate Analysis. J. Food Prot..

[9] Li H., Pan T., Li Y., Chen S., Li C. (2020). Functional principal component analysis for near-infrared spectral data: A case study on Tricholoma matsutakeis. Int. J. Food Eng..

[10] Li X., Wu Z., Xin F., Liu S., Yu X., Ma Q., Qiao Y. (2017). Quality-by-Design: Multivariate Model for Multicomponent Quantification in Refining Process of Honey. Pharmacogn. Mag..

[11] Santos D.D., Lima K.D., Cavalcante V., Coqueiro A., Consolin M.F.B., Filho N.C., Março P.H., Valderrama P. (2017). Multiproduct, Multicomponent and Multivariate Calibration: A Case Study by Using Vis-NIR Spectroscopy. Food Anal. Methods.

[12] Kalinin A.V., Krasheninnikov V.N., Sviridov A.P., Titov V.N. (2016). Near Infrared Spectrometry of Clinically Significant Fatty Acids Using Multicomponent Regression. J. Appl. Spectrosc..

[13] Wang P.P., Li Z., Qi T.T., Li S.-J., PAN S.-Y. (2015). Development of a method for identification and accurate quantitation of aroma compounds in Chinese Daohuaxiang liquors based on SPME using a sol–gel fibre. Food Chem..

[14] Wei Y., Zou W., Shen C.H., Yang J. (2020). Basic flavor types and component characteristics of Chinese traditional liquors: A review. J. Food Sci..

[15] Du L., He T., Li W., Wang R., Xiao D. (2015). Analysis of Volatile Compounds in Chinese Laobaigan Liquor using Headspace Solid-phase Microextraction Coupled with GC-MS. Anal. Methods.

[16] Zhang Z.Y., Sha M., Liu J., Wang H.-Y. (2017). Rapid quantitative analysis of Chinese Gu-Jing-Gong spirit for its quality control. J. Inst. Brew..

[17] Reich G. (2005). Near-infrared spectroscopy and imaging: Basic principles and pharmaceutical applications. Adv. Drug Deliv. Rev..

[18] Cui P., Zhao J., Liu M., Qi M., Wang Q., Li Z., Suo T., Li G. (2021). Non-invasive detection of medicines and edible products by direct measurement through vials using near-infrared spectroscopy: A review. Infrared Phys. Technol..

[19] Genisheva Z., Quintelas C., Mesquita D.P., Ferreira E., Oliveira J., Amaral A. (2018). New PLS analysis approach to wine volatile compounds characterization by near infrared spectroscopy (NIR). Food Chem..

[20] Vestia J., Barroso J.M., Ferreira H., Gaspar L., Rato A.E. (2018). Predicting calcium in grape must and base wine by FT-NIR spectroscopy. Food Chem..

[21] Fernández-Novales J., López M.I., Sánchez M.T., García J.-A., Morales J. (2008). A feasibility study on the use of a miniature fiber optic NIR spectrometer for the prediction of volumic mass and reducing sugars in white wine fermentations. J. Food Eng..

[22] Martelo-Vidal M.J., Vázquez M. (2015). Application of artificial neural networks coupled to UV–VIS–NIR spectroscopy for the rapid quantification of wine compounds in aqueous mixtures. CyTA-J. Food.

[23] Chen Q., Zhao J., Zhang H., Wang X. (2006). Feasibility study on qualitative and quantitative analysis in tea by near infrared spectroscopy with multivariate calibration. Anal. Chim. Acta.

[24] Ríos-Reina R., García-González D.L., Callejón R.M., Amigo J.M. (2018). NIR spectroscopy and chemometrics for the typification of Spanish wine vinegars with a protected designation of origin. Food Control.

[25] Li Z., Wang P.P., Huang C.C., Shang H., Pan S.-Y., Li X.-J. (2014). Application of Vis/NIR spectroscopy for Chinese liquor discrimination. Food Anal. Methods.

[26] Wei J., Zhou C., Han G., Via B., Swain T., Fan Z., Liu S. (2016). Classification and Identification of Plant Fibrous Material with Different Species Using near Infrared Technique—A New Way to Approach Determining Biomass Properties Accurately within Different Species. Front. Plant Sci..

[27] Sun Y., Chen L., Huang B., Chen K. (2017). A Rapid Identification Method for Calamine Using Near-Infrared Spectroscopy Based on Multi-Reference Correlation Coefficient Method and Back Propagation Artificial Neural Network. Appl. Spectrosc..

[28] Lin M., Mousavi M., Al-Holy M., Cavinato A.G., Rasco B.A. (2006). Rapid Near Infrared Spectroscopic Method for the Detection of Spoilage in Rainbow Trout (Oncorhynchus mykiss) Fillet. J. Food Sci..

[29] Anowar F., Sadaoui S., Selim B. (2021). Conceptual and empirical comparison of dimensionality reduction algorithms (PCA, KPCA, LDA, MDS, SVD, LLE, ISOMAP, LE, ICA, t-SNE). Comput. Sci. Rev..

[30] Lu C.Y., Feng J.S., Chen Y.D., Liu W., Lin Z., Yan S. Tensor Robust Principal Component Analysis: Exact Recovery of Corrupted Low-Rank Tensors via Convex Optimization. Proceedings of the 2016 IEEE Conference on Computer Vision and Pattern Recognition.

[31] Cai S.T., Luo Q.L., Yang M., Li W., Xiao M. (2019). Tensor Robust Principal Component Analysis via Non-Convex Low Rank Approximation. Appl. Sci..

[32] Driggs D., Becker S., Boyd-Graber J. (2019). Tensor Robust Principal Component Analysis: Better recovery with atomic norm regularization. arXiv.

[33] Bai J.S., Feng J.L. (2019). Robust Principal Component Analysis with Non-Sparse Errors. arXiv.

[34] Xu M.L., Yu Y., Ramaswamy H.S., Zhu S.M. (2017). Characterization of Chinese liquor aroma components during aging process and liquor age discrimination using gas chromatography combined with multivariable statistics. Sci. Rep..

[35] Stark E., Luchter K., Margoshes M. (1986). Near-Infrared Analysis (NIRA): A Technology for Quantitative and Qualitative Analysis. Appl. Spectrosc. Rev..

